# Supplementary material for: PvDBPII elicits multiple antibody-mediated mechanisms that reduce growth in a Plasmodium vivax challenge trial
Source: NPJ Vaccines. 2024 Jan 6;9:10. doi: 10.1038/s41541-023-00796-7 (PMC10771494; doi:10.1038/s41541-023-00796-7)
Supplement: Supplementary file 1 — Supplementary Figures [file 41541_2023_796_MOESM1_ESM.pdf]

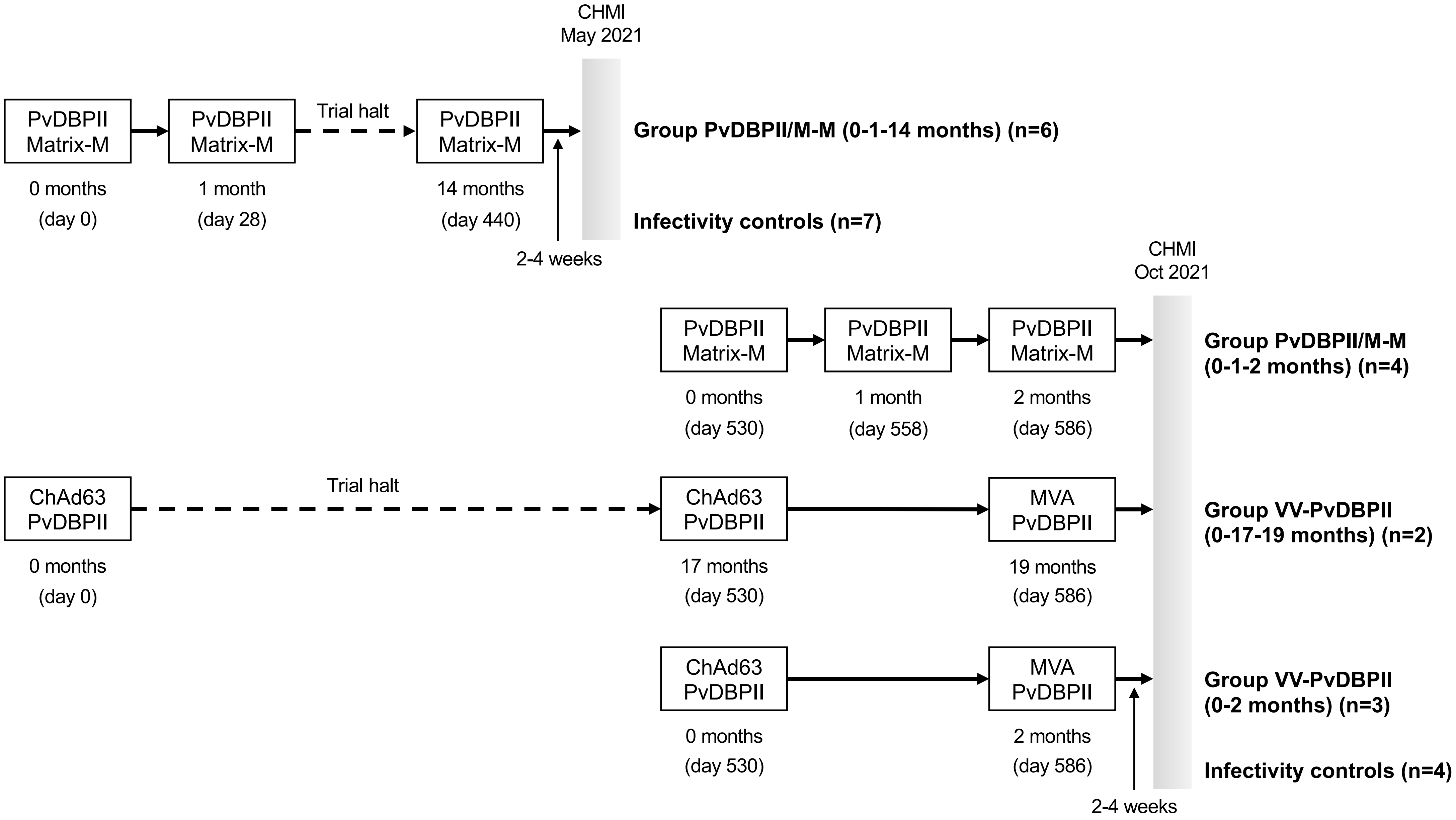

Supplementary Figure 1. Timing of immunizations over the course of the challenge trials.

Volunteers in the group PvDBPII/M-M (0-1-14 months) received the priming dose and first boost of recombinant PvDBPII formulated with Matrix-M^{TM} at 0 and 1 month (days 0 and 28) prior to trial halt. The delayed second boost was administered 14 months after priming dose (day 440). Volunteers in the group PvDBPII/M-M (0-1-14 months) (n = 6) underwent CHMI 2-4 weeks after the second boost in May 2021 along with infectivity controls (n = 7). Volunteers in the group PvDBPII/M-M (0-1-2 months) (n = 4) received PvDBPII/Matrix-M^{TM} at 0, 1 and 2 months (days 530, 558 and 586, respectively). Individuals in the group VV-PvDBPII (0-17-19 months) (n = 2) received ChAd63/PvDBPII before trial halt at day 0 and an additional ChAd63/PvDBPII followed by MVA/PvDBPII at 17 and 19 months (days 530 and 586). Volunteers in the group VV-PvDBPII (0-2 months) (n = 3) received ChAd63/PvDBPII and MVA/PvDBPII at 0 and 2 months (days 530 and 586). Groups PvDBPII/M-M (0-1-2 months), VV-PvDBPII (0-17-19 months) and VV-PvDBPII (0-2 months) underwent CHMI in October 2021 2-4 weeks after final boost, along with infectivity controls (n = 4).

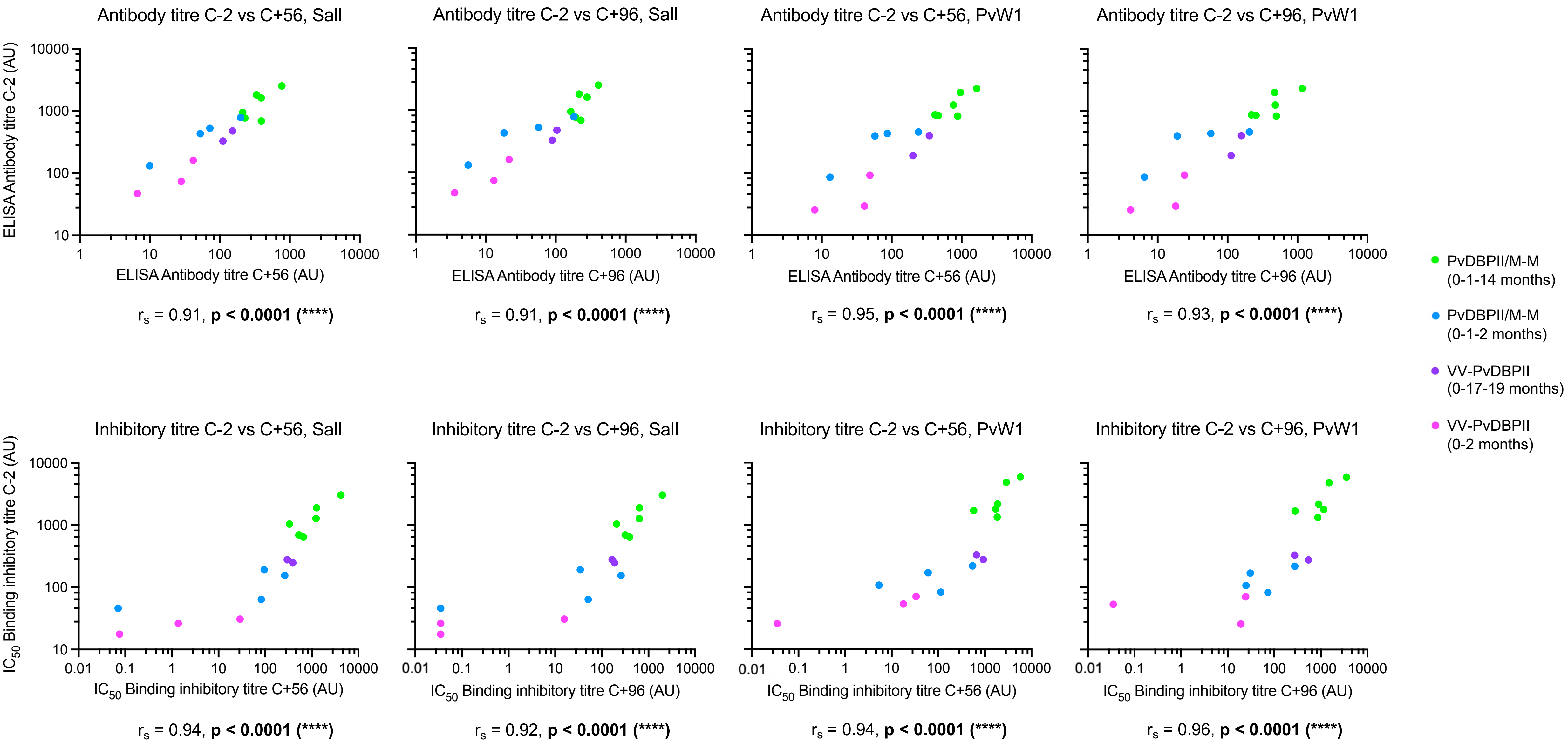

Supplementary Figure 2. Correlations between ELISA recognition titres and binding inhibition titres at C-2 and late time points.

Correlations between ELISA recognition titres and binding inhibition titres at C-2 and time points C+56 and C+96 were calculated for both PvDBPII variants SalI and PvW1 using Spearman's rank correlation tests. Correlations were also determined between binding inhibition titres at C-2 and time points C+56 and C+96 using Spearman's rank correlation tests. Correlation coefficients (r_s) and p values for each correlation are shown.

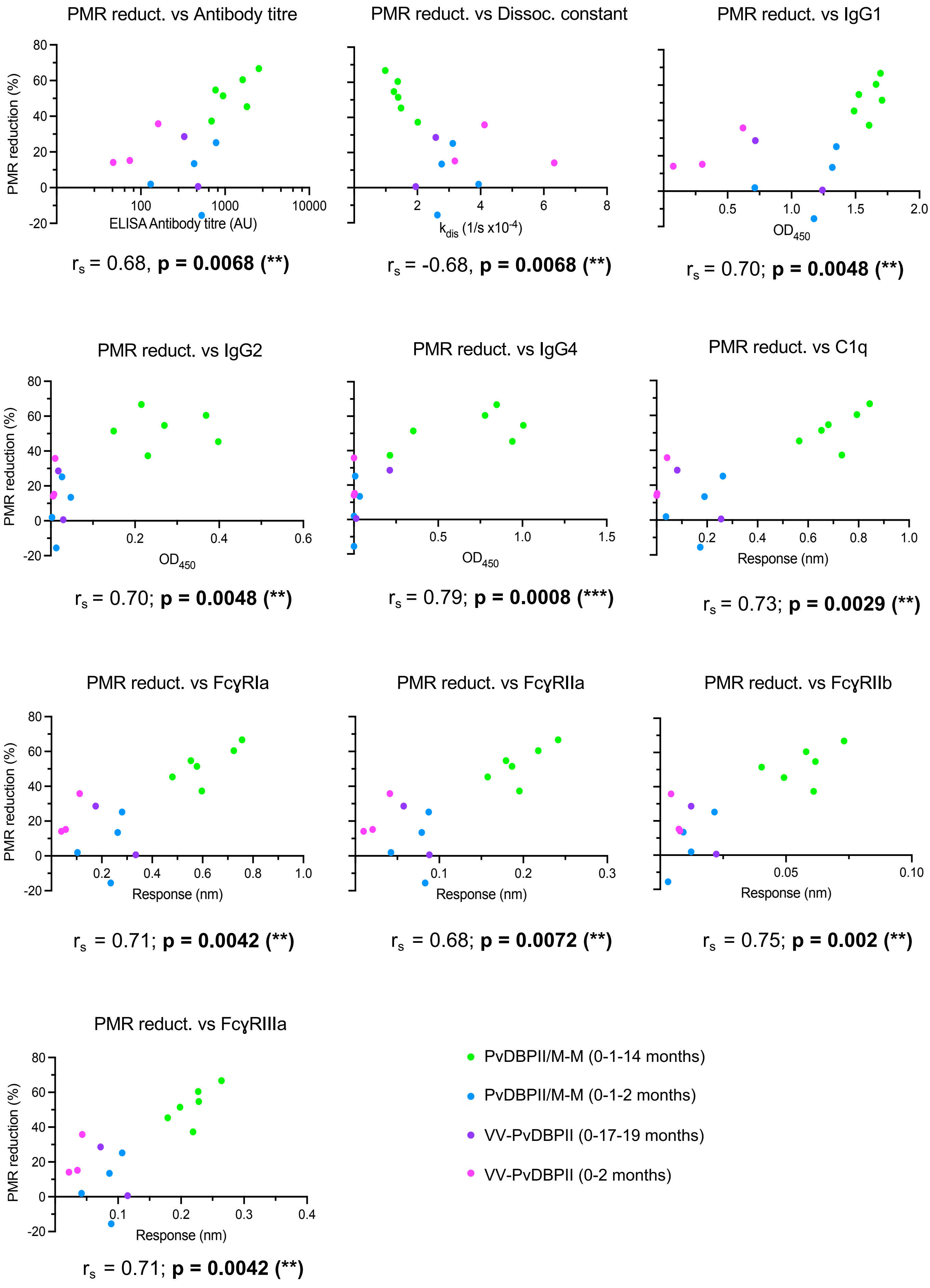

Supplementary Figure 3. Individual correlations of PMR reduction and features specific to PvDBPII SalI selected by the Boruta algorithm.

Correlations between PMR reduction and individual variables specific to PvDBPII SalI were calculated using Spearman's rank correlation tests. Correlation coefficients (r_s) and p values for each comparison are shown.

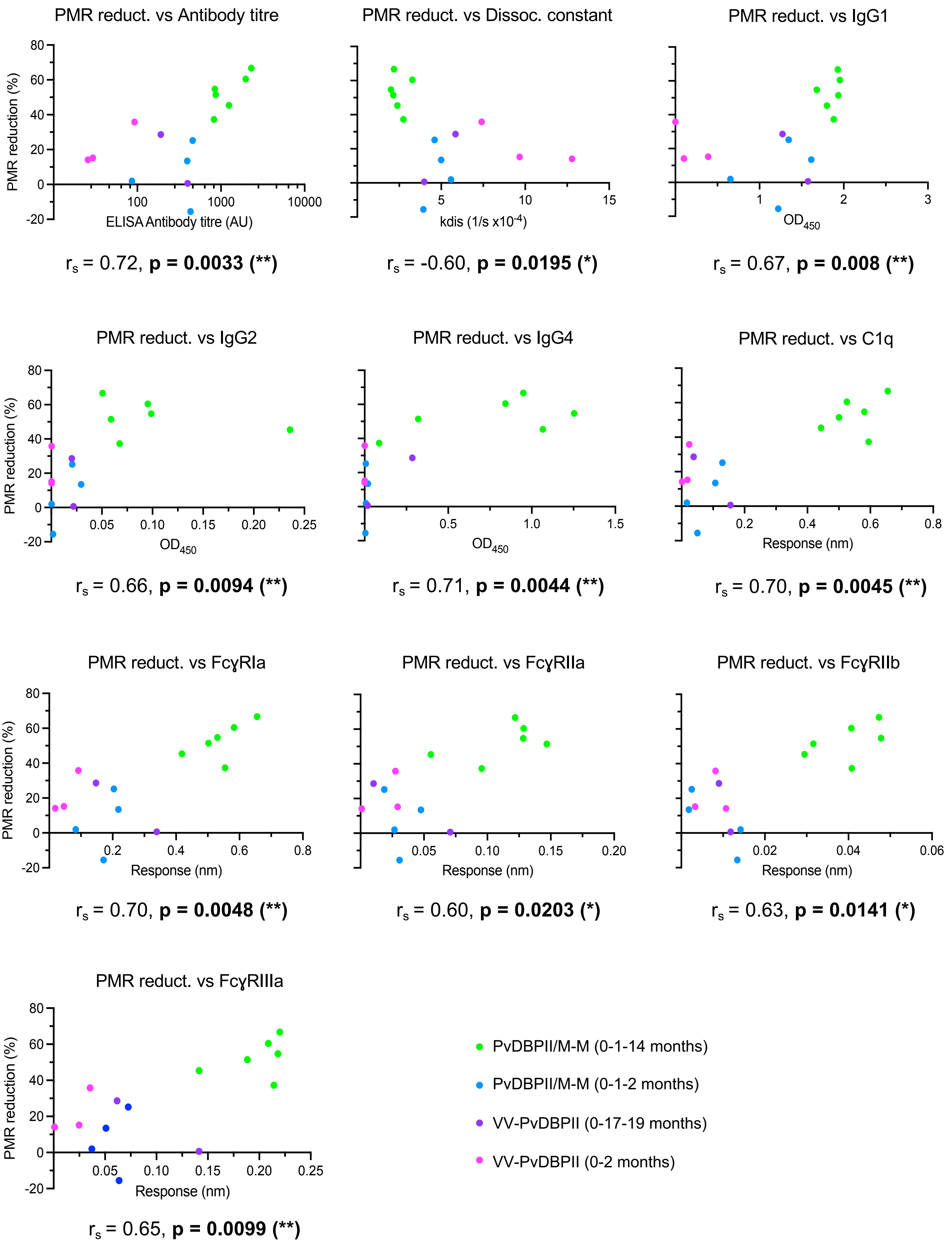

Supplementary Figure 4. Individual correlations of PMR reduction and features specific to PvDBPII PvW1 selected by the Boruta algorithm.

Correlations between PMR reduction and individual variables specific to PvDBPII PvW1 were calculated using Spearman's rank correlation tests. Correlation coefficients (r_s) and p values for each comparison are shown.

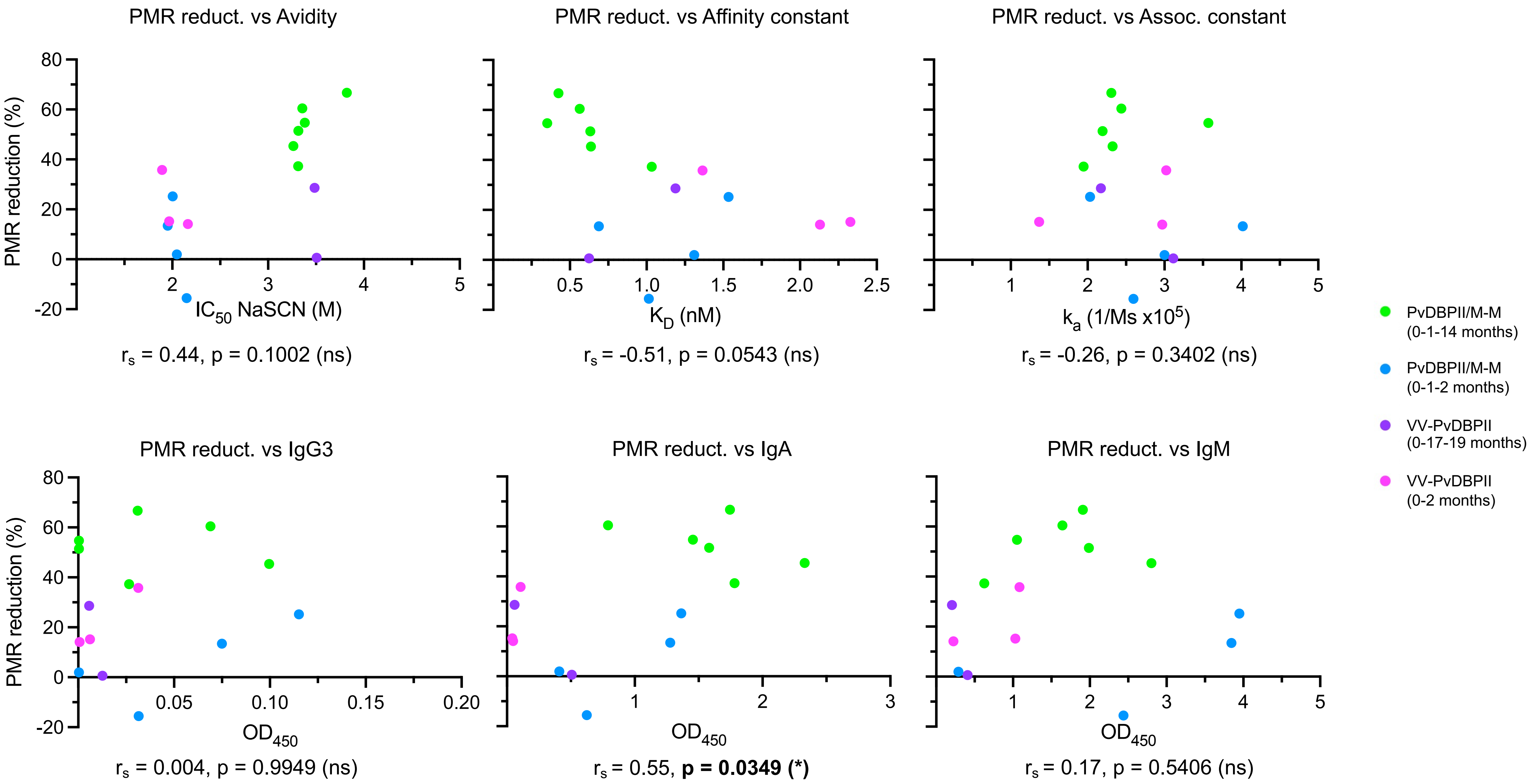

Supplementary Figure 5. Individual correlations of PMR reduction and features specific to PvDBPII SalI considered as unimportant by the Boruta algorithm.

Correlations between PMR reduction and individual variables specific to PvDBPII SalI were calculated using Spearman's rank correlation tests. Correlation coefficients (r_s) and p values for each correlation are shown, $*p < 0.05$.

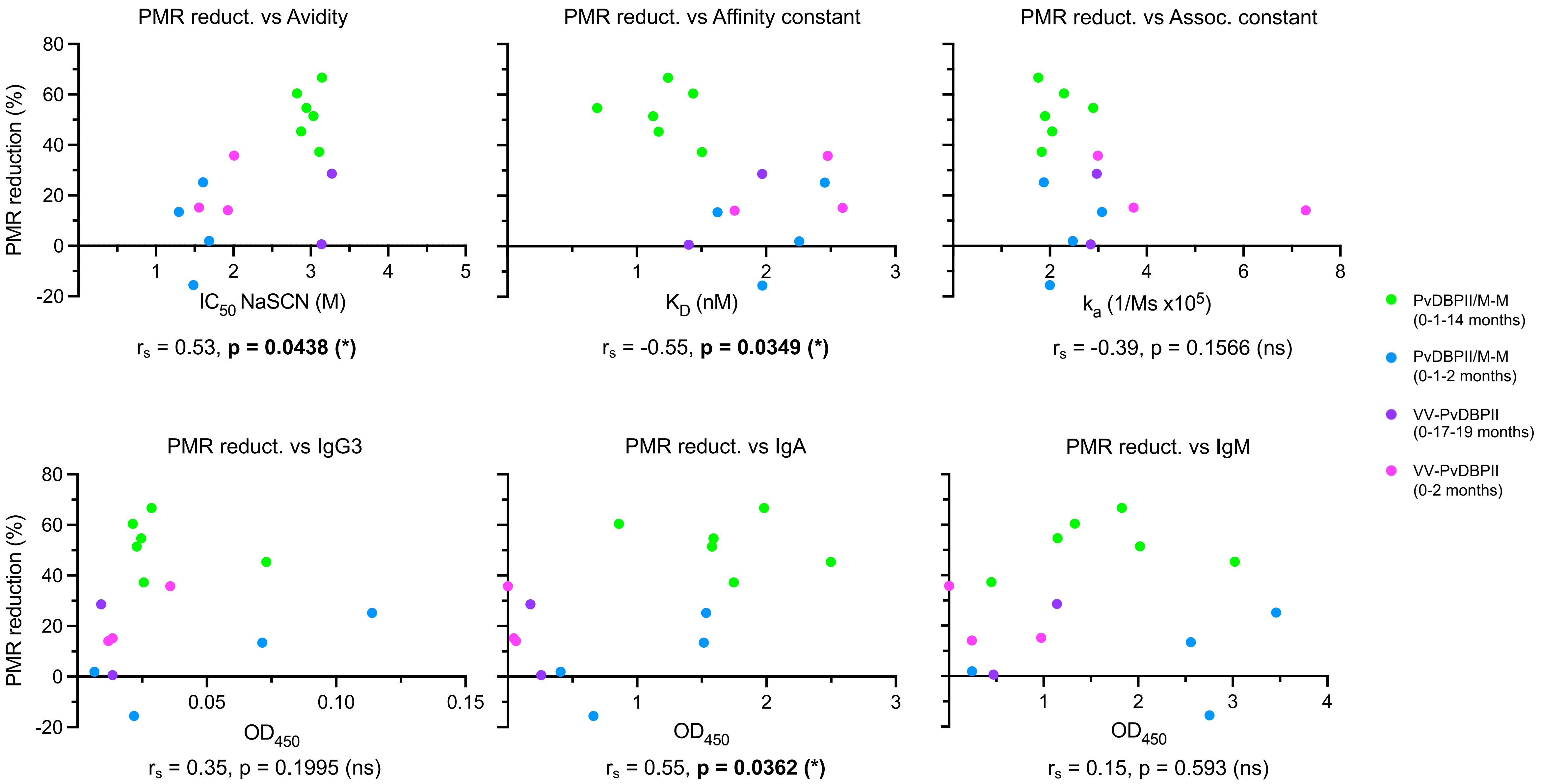

Supplementary Figure 6. Individual correlation of PMR reduction and features specific to PvW1 considered as unimportant by the Boruta algorithm. Correlation of PMR reduction and individual variables specific to PvDBPII PvW1 were calculated using Spearman's rank correlation tests. Correlation coefficients (r_s) and p values for each correlation are shown, *$p < 0.05$.

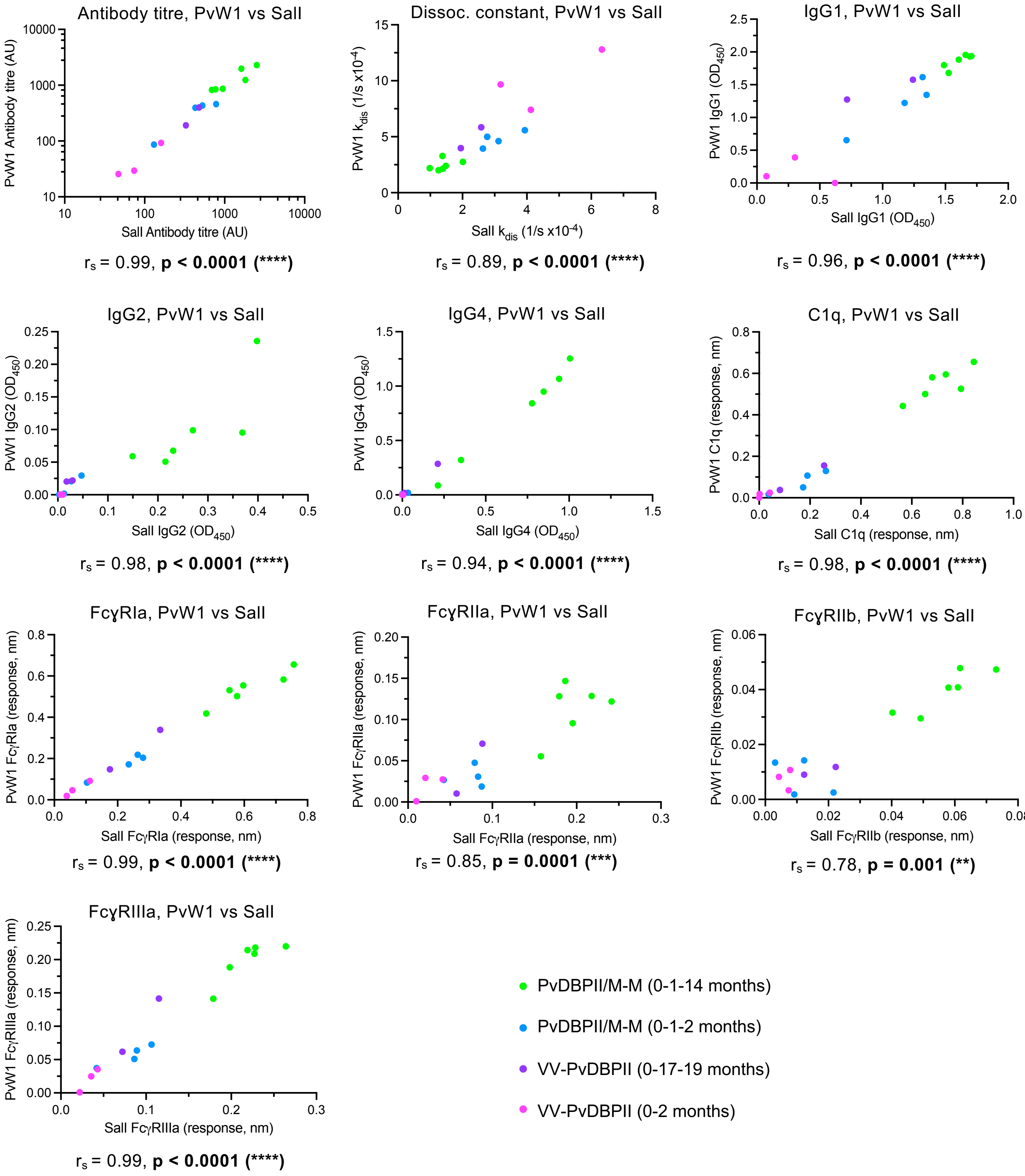

Supplementary Figure 7. Correlations between antibody functions selected by the Boruta algorithm specific to PvDBPII PvW1 and PvDBPII SalI.

Correlations between PvDBPII SalI-specific antibody functions and PvDBPII PvW1-specific antibody functions using Spearman's rank correlation tests. Correlation coefficients (r_s) and p values for each correlation are shown.
