# Supplementary material for: PvDBPII elicits multiple antibody-mediated mechanisms that reduce growth in a Plasmodium vivax challenge trial
Source: NPJ Vaccines. 2024 Jan 6;9:10. doi: 10.1038/s41541-023-00796-7 (PMC10771494; doi:10.1038/s41541-023-00796-7)
Supplement: Supplementary file 2 — REPORTING SUMMARY [file 41541_2023_796_MOESM2_ESM.pdf]

nature portfolio

Corresponding author(s): Chetan E Chitnis

Last updated by author(s): Oct 2, 2023

Reporting Summary

Nature Portfolio wishes to improve the reproducibility of the work that we publish. This form provides structure for consistency and transparency in reporting. For further information on Nature Portfolio policies, see our Editorial Policies and the Editorial Policy Checklist.

Statistics

For all statistical analyses, confirm that the following items are present in the figure legend, table legend, main text, or Methods section.

n/a	Confirmed	
☐	☒	The exact sample size (*n*) for each experimental group/condition, given as a discrete number and unit of measurement
☐	☒	A statement on whether measurements were taken from distinct samples or whether the same sample was measured repeatedly
☐	☒	The statistical test(s) used AND whether they are one- or two-sided<br>*Only common tests should be described solely by name; describe more complex techniques in the Methods section.*
☐	☒	A description of all covariates tested
☐	☒	A description of any assumptions or corrections, such as tests of normality and adjustment for multiple comparisons
☐	☒	A full description of the statistical parameters including central tendency (e.g. means) or other basic estimates (e.g. regression coefficient) AND variation (e.g. standard deviation) or associated estimates of uncertainty (e.g. confidence intervals)
☐	☒	For null hypothesis testing, the test statistic (e.g. *F*, *t*, *r*) with confidence intervals, effect sizes, degrees of freedom and *P* value noted<br>*Give P values as exact values whenever suitable.*
☒	☐	For Bayesian analysis, information on the choice of priors and Markov chain Monte Carlo settings
☐	☒	For hierarchical and complex designs, identification of the appropriate level for tests and full reporting of outcomes
☒	☐	Estimates of effect sizes (e.g. Cohen's *d*, Pearson's *r*), indicating how they were calculated

Our web collection on statistics for biologists contains articles on many of the points above.

Software and code

Policy information about availability of computer code

Data collection	n/a
Data analysis	Custom code. R version 4.2.2 and RStudio version 2022.12.0+353.

For manuscripts utilizing custom algorithms or software that are central to the research but not yet described in published literature, software must be made available to editors and reviewers. We strongly encourage code deposition in a community repository (e.g. GitHub). See the Nature Portfolio guidelines for submitting code & software for further information.

Data

Policy information about availability of data

All manuscripts must include a data availability statement. This statement should provide the following information, where applicable:

- Accession codes, unique identifiers, or web links for publicly available datasets
- A description of any restrictions on data availability
- For clinical datasets or third party data, please ensure that the statement adheres to our policy

All data that support the findings of this study will be made available online with the manuscript. The underlying code for this study is not publicly available but may be made available to qualified researchers on reasonable request from the corresponding author.

Research involving human participants, their data, or biological material

Policy information about studies with human participants or human data. See also policy information about sex, gender (identity/presentation), and sexual orientation and race, ethnicity and racism.

Reporting on sex and gender	Data on sex and gender are not relevant for the study and are not reported.
Reporting on race, ethnicity, or other socially relevant groupings	Data on race, ethnicity or any other social relevant groups are not relevant for the study and are not reported.
Population characteristics	The age of volunteers is between 19 and 48 years old. Volunteers were positive for the Duffy antigen receptor for chemokines (DARC).
Recruitment	The Univerisity of Oxford recruited participants who voluntarily decided to enroll in the clinical trial. Volunteers who met inclusion criteria were enrolled.
Ethics oversight	The study was approved by UK National Health Service Research Ethics Services.

Note that full information on the approval of the study protocol must also be provided in the manuscript.

Field-specific reporting

Please select the one below that is the best fit for your research. If you are not sure, read the appropriate sections before making your selection.

☒ Life sciences ☐ Behavioural & social sciences ☐ Ecological, evolutionary & environmental sciences

For a reference copy of the document with all sections, see nature.com/documents/nr-reporting-summary-flat.pdf

Life sciences study design

All studies must disclose on these points even when the disclosure is negative.

Sample size	The original sample size plan was 15 volunteers per group ihowever due to Covid-19 pandemic the trial was delayed and several volunteers could not complete the trial. The final sample size was 26 distributed in different groups.
Data exclusions	No data was excluded.
Replication	Replications of the results were successful.
Randomization	Trial groups were randomized. However, delays caused by Covid-19 pandemic resulted in withdrawl of volunteers from the study.
Blinding	Investigators were blinded when assesing efficacy of the vaccine candidates. Efficacy was determined as per the blood-stage parasitemia measured by qPCR in vaccine groups and infectivity controls.

Reporting for specific materials, systems and methods

We require information from authors about some types of materials, experimental systems and methods used in many studies. Here, indicate whether each material, system or method listed is relevant to your study. If you are not sure if a list item applies to your research, read the appropriate section before selecting a response.

Materials & experimental systems

n/a	Involved in the study	
☐	☒	Antibodies
☐	☒	Eukaryotic cell lines
☒	☐	Palaeontology and archaeology
☐	☒	Animals and other organisms
☐	☒	Clinical data
☒	☐	Dual use research of concern
☒	☐	Plants

Methods

n/a	Involved in the study	
☒	☐	ChIP-seq
☒	☐	Flow cytometry
☒	☐	MRI-based neuroimaging

Antibodies

Antibodies used	Anti-PvDBPII rabbit sera: home made.

Antibodies used	Anti-PvDBPII human sera: derived from a previous PvDBPII-vaccine Phase I trial.<br>Peroxidase-conjugated anti-rabbit IgG secondary antibody (A6154, Sigma, lot SLBV9141).<br>Peroxidase-conjugated anti-human IgG rabbit antibodies (A8792, Sigma, lot 109M4892V).<br>Alexa Flour 488-conjugated goat anti-human IgG (H+L) secondary antibodies (A11013, Themo Fisher, lot 49018A).<br>Biotin-conjugated mouse monoclonal anti-human IgG1 (B6775, Life Technologies, clone 8c/6-39, batch 0000124411).<br>Biotin-conjugated mouse monoclonal anti-human IgG2 (B3398, Life Technologies, clone HP-6014, lot 069M4888V).<br>Biotin-conjugated mouse monoclonal anti-human IgG3 (B3623, Sigma, clone HP-6050, batch 000082484).<br>Biotin-conjugated mouse monoclonal anti-human IgG4 (B3648, Sigma, clone HP-6025, batch 0000113950).<br>Peroxidase-conjugated goat polyclonal anti-human IgA alpha-chain (A0295, Sigma, batch 0000119777).<br>Peroxidase-conjugated goat polyclonal anti-human IgM mu-chain (401905, Millipore, lot 3781087).
Validation	Anti-PvDBPII rabbit sera (home made) was validated to bind PvDBPII recombinant protein by ELISA.<br>Anti-PvDBPII human sera was derived from a previous PvDBPII-vaccine Phase I trial and it was validated in the same study (PMID: 30302285).<br>Biotin-conjugated mouse monoclonal anti-human IgG1 was validated for specificity of human immunoglobulins by the manufacturer using immunocytochemistry.<br>Biotin-conjugated mouse monoclonal anti-human IgG2 was validated for specificity of human immunoglobulins by the manufacturer using ELISA.<br>Biotin-conjugated mouse monoclonal anti-human IgG3 was validated for specificity of human immunoglobulins by the manufacturer using ELISA.<br>Biotin-conjugated mouse anti-human IgG4 was validated for specificity of human immunoglobulins by the manufacturer using ELISA.<br>Peroxidase-conjugated goat polyclonal anti-human IgA alpha-chain was validated for specificity of human immunoglobulins by the manufacturer using ELISA and immunohistochemistry.<br>Peroxidase-conjugated goat polyclonal anti-human IgM mu-chain was validated for specificity by manufacturer using immunoelectrophoresis against normal human serum.

Eukaryotic cell lines

Policy information about cell lines and Sex and Gender in Research

Cell line source(s)	cell-line HEK293T (CRL-3216, ATCC).
Authentication	Cell lines ordered from ATCC.
Mycoplasma contamination	Cell lines ordered from ATCC.
Commonly misidentified lines<br>(See ICLAC register)	None.

Animals and other research organisms

Policy information about studies involving animals; ARRIVE guidelines recommended for reporting animal research, and Sex and Gender in Research

Laboratory animals	No laboratory animals were used in this study.
Wild animals	None.
Reporting on sex	*Indicate if findings apply to only one sex; describe whether sex was considered in study design, methods used for assigning sex. Provide data disaggregated for sex where this information has been collected in the source data as appropriate; provide overall numbers in this Reporting Summary. Please state if this information has not been collected. Report sex-based analyses where performed, justify reasons for lack of sex-based analysis.*
Field-collected samples	*For laboratory work with field-collected samples, describe all relevant parameters such as housing, maintenance, temperature, photoperiod and end-of-experiment protocol OR state that the study did not involve samples collected from the field.*
Ethics oversight	*Identify the organization(s) that approved or provided guidance on the study protocol, OR state that no ethical approval or guidance was required and explain why not.*

Note that full information on the approval of the study protocol must also be provided in the manuscript.

Clinical data

Policy information about clinical studies

All manuscripts should comply with the ICMJE guidelines for publication of clinical research and a completed CONSORT checklist must be included with all submissions.

Clinical trial registration	The trials are registered under the following ClinicalTrials.gov numbers: NCT03797989 (trial VAC069), NCT04009096 (trial VAC071) and NCT04201431 (trial VAC079).
Study protocol	Study protocol is not accesible to anyone other than the Sponsor, Ethics Committee and Investigators as it contains confidential information.

Data collection	Study location: Oxford, Oxfordshire, United Kingdom, OX3 7LE. Centre for Clinical Vaccinology and Tropical Medicine.<br>Recruitment 2019-07/2021-06.<br>Data collection 2019-09/2022-08
Outcomes	Primary outcomes: to assess safety and tolerability of the vaccines by actively and passively collecting data on adverse events; to assess the reduction of the parasite multiplication rate in vaccinated subjects compared to controls by qPCR.<br>Secondary outcomes:to assess the immunogenicity of the vaccines by a variety of immunological assays: ELISA antibody titers, avidity, isotyping, etc; to assess the immunological readouts for association with a reduced parasite multiplication rate by multiple statistical tests: Random Forest, Spearman correlation, Boruta algorithm, etc.
